# Clinical, laboratory, and orbital imaging features of giant cell arteritis in comparison to non-arteritic anterior ischemic optic neuropath: a single center case series

**DOI:** 10.3389/fopht.2024.1498968

**Published:** 2024-12-24

**Authors:** Rami W. Eldaya, Yi-Hsien Yeh, Leanne Stunkel, Matthew S. Parsons, Gregory P. Van Stavern

**Affiliations:** ^1^ Department of Neuroradiology, The University of Texas MD Anderson Cancer Center, Houston, TX, United States; ^2^ Department of Ophthalmology and Visual Sciences, Washington University School of Medicine, St. Louis, MO, United States; ^3^ Department of Ophthalmology & Visual Sciences, School of Medicine, Washington University in St. Louis, St Louis, MO, United States; ^4^ Department of Neurology, School of Medicine, Washington University in St. Louis, St. Louis, MO, United States; ^5^ Mallinckrodt Institute of Radiology, Washington University School of Medicine, St Louis, MO, United States

**Keywords:** MRI, giant cell (temporal) arteritis, NAION, optic nerve (ON), optic neuritis (ON)

## Abstract

**Background:**

Giant cell arteritis (GCA) is the most common vasculitis in patients older than 50 years and is considered a “do not miss” diagnosis. However, it remains a diagnostic challenge given overlapping clinical syndromes such as non-arteritic anterior ischemic optic neuropathy (NAION) and poorly explored imaging findings.

**Materials and methods:**

In this retrospective study between the time period of January 2013 and December 2021, a total of 13 consecutive patients with a pathological diagnosis of GCA and 8 patients with clinical diagnosis of NAION were isolated. Demographic and clinical data for each patient were collected, including pertinent laboratory data. Pertinent physical exam data was also collected, including fundoscopic exam and visual acuity. Two neuroradiologist assessed the orbital MRI imaging findings of GCA and NAION for the presence and characterization of imaging abnormalities. Assessment for potential relationship between GCA orbital findings, laboratory and visual outcomes was performed. Finally, comparison between GCA and NAION imaging findings was performed.

**Results:**

13 GCA patients were assessed. 9 patients had abnormal orbital findings. Of these 8 patients had bilateral orbital involvement The most common imaging findings was perineuritis of the optic nerve sheath, present in 7 patients. In total, 8 NAION patients were assessed. All patients demonstrate optic nerve involvement. The Snellen test was converted to logmar, and visual acuity was assessed for both NAION and GCA for each eye at diagnosis and at the last follow-up. There was no statistical significance for either eye for both GCA and NAION at initial diagnosis and final follow-up. In the 4 GCA patients with normal MRI findings and 9 GCA patients with abnormal MRI findings, there was no statistical significance between initial presentation and final follow-up visual acuity.

**Conclusion:**

GCA and NAION are potentially overlapping clinical syndromes with different treatment approach and poorly explored imaging findings. Our case series assesses the orbital imaging findings of both syndromes while noting different imaging pattern of both on MRI, which can serve as a potential tool to aid in diagnosis of both.

## Introduction

Giant cell arteritis (GCA) is the most common vasculitis in patients older than 50 and is considered a “do not miss” diagnosis, given the risk of rapid permanent vision loss in up to 20% of untreated cases (1–3). However, its diagnosis remains challenging, given varied clinical presentations, overlapping diseases, and a lack of consensus criteria for clinical diagnosis (4, 5).

Multiple laboratory and clinical features can suggest GCA, but no single feature is diagnostic (5). However, combining laboratory and clinical findings can help guide further investigation, including biopsy and subsequent imaging (4, 6).

Temporal artery biopsy has long been considered the gold standard for diagnosing GCA (3). However, yield and sensitivity remain low due to multiple challenges, including the stringent recommended sample size, the failure of ultrasound guidance to improve biopsy sensitivity, the large variability between pathologists in interpreting the biopsy, and the variation in temporal artery anatomy (3, 7–15).

Imaging of GCA has historically focused on identifying large vasculitis, detecting temporal artery vasculitis, guiding biopsy sites, and detecting intracranial and systemic complications. Ultrasound has been studied extensively for detecting temporal artery vasculitis and aiding in biopsy site identification, with mixed results for both (11, 16–19). Computed tomography (CT), computed tomography angiogram (CTA), and positron emission tomography (PTE/PET/CT) have been assessed in the detection of large vessel vasculitis in GCA (20–24). In addition to imaging of large vessels vasculitis changes and intracranial complication of GCA, magnetic resonance imaging/angiography (MRI/MRA) through vessel wall imaging offers the ability to assess and detect smaller vessels vasculitis including temporal artery, further aiding in diagnosis and biopsy guidance (25–28).

Despite the extensive imaging data for assessing vasculitis in GCA, there is significantly limited data in assessing orbital findings in patients with GCA, and little is known regarding the presence and significance of such findings. Furthermore, most published case reports and series focused on assessing the optic nerve sheath complex and papilledema (29–31). In this case series, we assess the intra-orbital imaging findings of 13 patients with a confirmed diagnosis of GCA. We hypothesize that intra-orbital MRI findings in GCA are more prevalent than believed. Furthermore, we assess the relationship between imaging findings, symptoms, and laboratory markers. We also observe the relationship between imaging findings and long-term vision outcomes. Finally, we compare orbital MRI imaging findings of GCA-associated arteritic anterior ischemic optic neuropathy (A-AION) and non-arteritic anterior ischemic optic neuropathy (NAION), a clinically overlapping entity with different management and disease course, to assess orbital MRI potential as an aiding diagnostic tool in distinguishing between the two entities. Arteritic anterior ischemic optic neuropathy is the most common cause of visual loss in GCA (32), and deciding whether the patient has the arteritic or non-arteritic form of the disease is often challenging.

## Materials and methods

The institutional review board at Washington University School of Medicine in Saint Louis (WashU) approved this retrospective study.

### Patient selection and inclusion criteria

Between January 2013 and December 2021, 13 consecutive patients with a diagnosis of GCA and eight patients with NAION were identified. Assessment of pathology, clinical data, and imaging data was then performed to refine the data with the following inclusion criteria:

#### GCA

1. Tissue diagnosis of GCA via temporal artery biopsy performed or confirmed at WashU 2. Clinical diagnosis of GCA, 3. Orbital and/or brain MRI is available in PACS for review. All these three criteria must be met, and the lack of any of these criteria excludes the patient from the study.

#### NAION

1. Clinical diagnosis of NAION, 2. Orbital and/or brain MRIs are available in PACS for review. Both criteria must be met, and the patient is only included in the study if they are met.

### Clinical and laboratory data

Patients’ electronic medical records were accessed, and demographic and clinical data for each patient were collected, including age at diagnosis, gender, presenting symptoms, available imaging, and pertinent laboratory data (C-reactive protein and erythrocyte sedimentation rate, ESR). Pertinent physical exam data, including fundoscopic exams and visual acuity, were also collected. Lastly, the patient’s vision status was documented at the last available follow-up. A fellowship-trained neuro-ophthalmologist confirmed the diagnosis of AION (both arteritic and non-arteritic).

### Imaging review

All MRI characteristics were reviewed by two fellowship-trained neuroradiologists with 4 years.’

(R.E.) and 17 years’ experience (M.P). Both readers were blinded to the radiology reports, clinical data, and each other’s interpretation. Initially, each neuroradiologist assessed the imaging for abnormalities, which was used to calculate Interobserver agreement. Subsequently, both neuroradiologists reviewed the images together while blinded to the radiology report and resolved disagreements with consensus.

### MR orbits/brain imaging review

All MRI brain and orbits were acquired on 1.5T or 3T Siemens Scans. Brain MRI exams included a 4 mm Sagittal T1WI, 5 mm axial T2WI, axial 5 mm fluid-attenuated inversion recovery (FLAIR), 5 mm axial and 3 mm coronal diffusion-weighted images (DWI), 5 mm axial post-contrast T1WI, and 5 mm fat, saturated coronal post-contrast T1WI. Orbits MRI protocol included 3 mm axial T1WI of the orbits, 0.7 mm axial constructive interference in steady state (CISS) of the orbits, 4 mm coronal fat-saturated FLAIR of the orbits, 1 mm axial post-contrast volumetric interpolated breath-hold examination (VIBE) of the orbits, 4 mm coronal spectral attenuated inversion recovery (SPAIR) of the orbits.

All MR orbits and/or brain images were analyzed to assess bilateral orbital structures for abnormalities. Optic disc edema was assessed as present or absent on fluid-sensitive sequences and diffusion-weighted images in cases with clinical optic disc edema (33). Optic nerve sheath complex enhancement and/or signal abnormality of fluid-sensitive sequences, optic nerve segment abnormality location, and enhancement pattern were recorded. Involvement of extraocular muscles and globe was documented. Lastly, a comparison of orbital findings between GCA and NAION was performed.

### Statistical analysis

The mean and standard deviations of pertinent laboratory data were calculated and reported in the result section. Also, the interquartile range (IQR) was calculated when indicated. An interobserver agreement, Cohen Kappa, was calculated for the presence of orbital abnormality on MRI for both GCA and NAION. Disagreements were resolved by consensus agreement.

Association between clinical symptoms and imaging abnormality was noted, and statistical significance was calculated using a paired t-test when feasible. Similarly, the association between laboratory abnormality and the presence of imaging abnormality for GCA patients was noted, and when possible, statistical significance was calculated using a paired t-test. A comparison of orbital imaging findings between GCA and NAION was performed to assess potential imaging markers that can distinguish between the two entities. Visual acuity utilizing Snellen visual charts was standardized into logmar format to allow for statistical analysis as described previously (34, 35). A comparison of visual acuity at presentation and final follow-up for both GCA and NAION was performed using a paired t-test. Lastly, an exploratory observation of the association between abnormal imaging findings and long-term visual outcomes was documented, and statistical significance was performed utilizing a paired t-test.

## Results

Clinical data for both GCA and NAION is summarized in **Table 1**. In total, 13 patients (7 females) with biopsy-proven GCA and 8 NAION patients (6 females) were included. Patients with NAION were younger, which was statistically significant. There was no significant difference between the two populations regarding symptoms to presentation and symptoms to MRI. As expected, ESR and CRP were higher in GCA patients, which was statistically significant, while there was a trend for higher platelets in GCA patients (0.051). Only two patients with NAION had temporal artery biopsies. One patient had a biopsy 3 days after presentation and another 47 days after presentation.

**Table 1:** Clinical characteristics of both GCA (with and without AION) and NAION.

Characteristic (n)	GCA	NAION	P value*
Patient population (females)	13 (7)	8 (6)	
Presenting symptoms (number)	12 visual loss 7 headaches 7 jaw claudication 6 scalp tenderness 3 weight loss 1 temporal artery induration 1 no symptoms	8 Vision loss or field defect	
Optic disc edema	9 present 3 normal 1 not reported	7 present 1 not reported	
Abnormal fundoscopic exam	7 abnormal 6 normal	3 abnormal 4 normal 1 not examined	
Mean age in years at presentation (SD)	76.31 (7.1)	60.75 (7.12)	0.00027*
Symptoms to presentation in days	7.91	15.63	0.13
Symptoms to MRI in days	9.33	25.14	0.16
Presentation to biopsy in days	3.38	25¹	
ESR Mean ESR Median and IQR	57.62 52 (37.5-76.5)	12.86 12 (7-16)	0.0039*
CRP Mean CRP Median and IQR	57.74 41.5 (17.561.85)	1.27 0.8 (0.41.4)	0.039*
Platelets Mean Platelets Median and IQR	363.1 389 (278.5-443)	267.43 270 (207299)	0.051
Follow up in months Mean Follow up Median and IQR	20.62 12 (1.5-33)	10.14 10 (5-13)	

*P value <0.05 denotes statistical significance.

Only two patients with NAION had biopsies. A patient after 3 days and another after 47 days from presentation.

### MRI imaging findings

#### GCA

**Figure 1:**
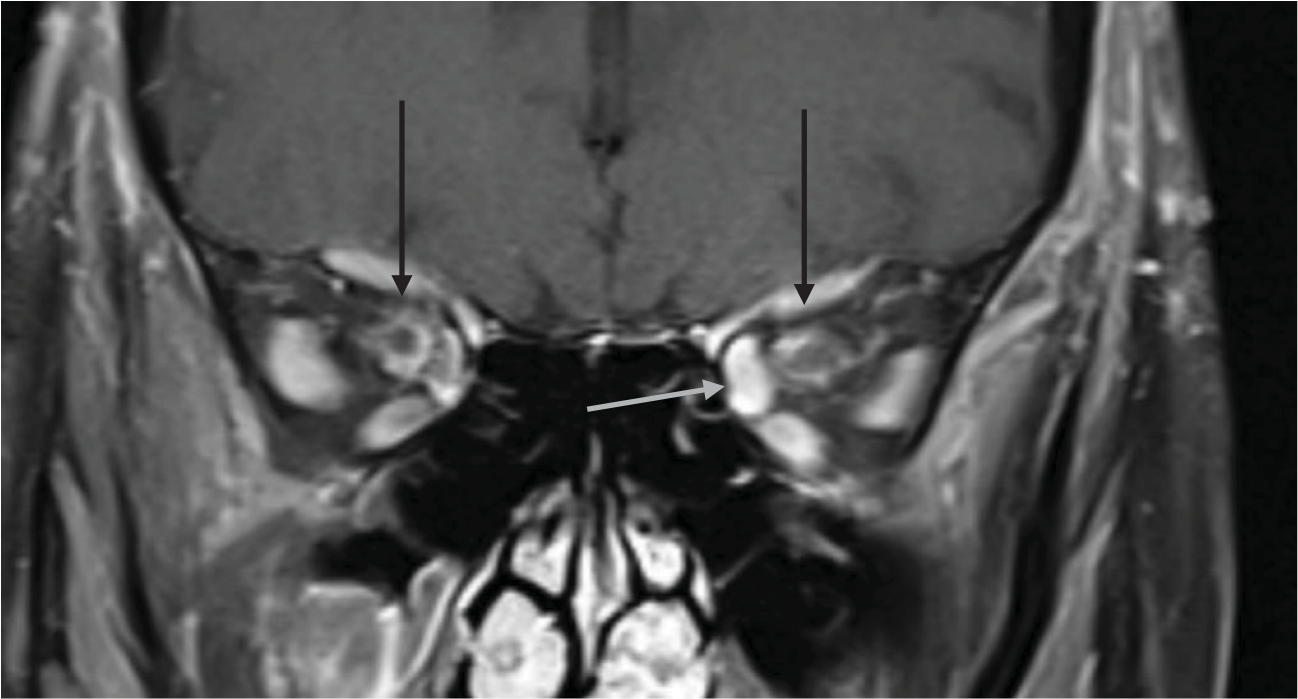
74-year-old presenting with acute left eye vision loss (OS) with jaw claudication, scalp tenderness, and headaches. Fundoscopic exam demonstrated grade 3 OS optic disc edema with heme. Subsequent biopsy confirmed GCA. Coronal fat-saturated post-contrast sequence demonstrates enhancement of the bilateral intraorbital optic nerves sheath suggestive of perineuritis (black arrows). The left medial rectus muscle is asymmetrically thickened and enhancing compared to the right medial rectus muscle, suggesting myositis (gray arrow).

**Figure 2:**
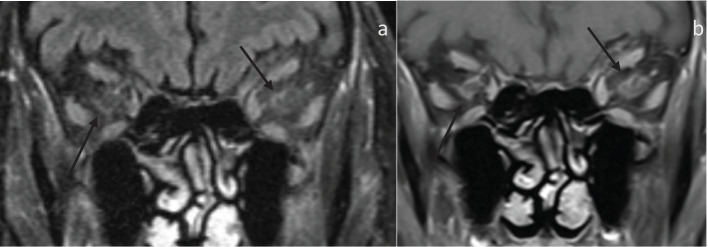
80-year-old presenting with acute left eye vision loss (OS) with jaw claudication, headache, and weight loss. A fundoscopic exam demonstrated left optic disc edema. Subsequent biopsy confirmed GCA. **(A)** Coronal fat-saturated FLAIR demonstrates FLAIR hyperintensity along the bilateral intraorbital optic nerves sheath (arrows), suggestive of optic perineuritis. **(B)** The coronal fat-saturated post-contrast sequence demonstrates enhancement of the bilateral intraorbital optic nerve sheath suggestive of perineuritis (arrows).

In total, 13 patients and 26 orbits were assessed, data summarized in **Table 2**. 9 patients had abnormal orbital findings (69.2%). Of these eight patients (88.89%), bilateral orbital involvement was observed on imaging, including four patients with visual symptoms involving only one orbit. The most common imaging findings was perineuritis of the optic nerve sheath complex detected on MRI as enhancement along the optic nerve sheath confined to the intraorbital segment in all cases (**Figures 1**, **2**). There was no enhancement or hyperintensity of the optic nerve on fluid-sensitive sequences in any case. Bilateral perineuritis was present in 7 patients. Two patients had imaging evidence of optic disc edema correlating with fundoscopic findings. In both cases, there was perineuritis. 2 patients had isolated left medial rectus muscle involvement, characterized by thickening and enhancement of the muscle compared to the contralateral side. In one patient, this was the only abnormal imaging finding.

**Table 2:** GCA (with and without AION) and NAION MRI orbital imaging findings.

Characteristic (number of patients)	
MR orbit findings of GCA	4 normal 9 abnormal - 8 perineuritis of the intra-orbital segment o 7 bilateral - 2 optic disc edema o 1 bilateral - 2 medial rectus muscle thickening and enhancement
MR orbit findings of NAION	8 abnormal - 8 unilateral optic nerve T2/FLAIR hyperintensity o 7 intra-orbital segment o 1 intra-orbital and intracanalicular segments - 2 bilateral papilledema

The inter-reliability agreement between both readers for the detection of orbital abnormality was calculated using Cohen’s Kappa coefficient, which was 0.491, suggesting moderate agreement.

#### NAION

**Figure 3:**
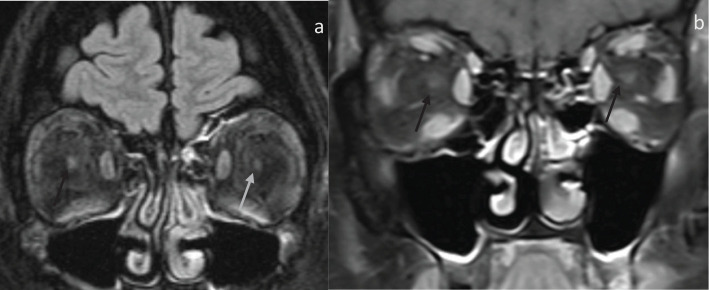
53-year-old presenting with sequential horizontal field vision loss with fundoscopic exam demonstrating bilateral grade 4 optic disc edema. Clinical diagnosis was consistent with NAION. **(A)** Coronal fat-saturated FLAIR demonstrates FLAIR hyperintensity and indistinctness of the right intraorbital optic nerve (black arrow) compared to the left optic nerve (gray arrow). **(B)** Coronal fat, saturated post-contrast, demonstrates no enhancement within both intraorbital optic nerves (arrows).

8 patients and 16 orbits were assessed, as shown in **Table 2**. All patients demonstrate optic nerve involvement, suggested by an increased signal within the optic nerve on fluid-sensitive sequences. However, none of the involved optic nerves demonstrated any associated enhancement (**Figure 3**). The optic nerve signal abnormality was confined to the intraorbital segment in 7 patients, and in 1 patient, it extended to the intracanalicular segment. Two patients had bilateral optic disc edema on imaging. Interestingly, all patients had unilateral optic nerve involvement involving the symptomatic side. In cases of sequential visual loss, the initial site of symptoms was the site that demonstrated the optic nerve signal abnormality.

The inter-reliability agreement between both readers for detecting orbital abnormality was calculated utilizing Cohen’s Kappa coefficient, which was 0.875, suggesting almost perfect agreement.

### Visual acuity comparison

The Snellen test was converted to logmar, and visual acuity was assessed for both NAION and GCA in each eye at diagnosis and the last follow-up. There was no statistical significance for either eye for both GCA and NAION at initial diagnosis and final follow-up (**Table 3**).

**Table 3:** Logmar visual acuity comparison for NAION and GCA at initial presentation and final follow-up.

Pathology (number of patients)	Initial presentation (mean)	Last follow up (mean)	P value*
NAION OD (8)	0.45	0.29	0.46
NAION OS (8)	0.55	0.74	0.15
GCA OD (13)	1.281538	1.476923	0.7
GCA OS (13)	0.901538	0.844615	0.87
GCA normal MRI findings (4)	1.038	1.202	0.53
GCA abnormal MRI findings (9)	1.125	1.135	0.96

*P value <0.05 denotes statistical significance.

Subsequently, this was further sub-analyzed to assess patients with GCA with and without associated abnormal MRI findings. There was no statistical significance between the four patients with normal MRI findings between the initial presentation and final follow-up visual acuity, **Table 3**. Similarly, there was no statistical significance between the nine patients with abnormal MRI findings between the initial presentation and final follow-up visual acuity, **Table 3**. Lastly, an exploratory attempt was made to assess the potential association between imaging findings, initial visual acuity, and subsequent outcomes. A comparison between the visual acuity of patients with normal and abnormal MRI orbit imaging findings was performed at the initial and final presentations, which demonstrated no statistical significance, **Table 4**. This was also correlated with comparing laboratory tests for both subgroups at presentation. There was no statistical significance in ESR (p = 0.35), CRP (p = 0.36), or platelets level (0.14) between GCA patients with positive and negative orbital MR findings.

**Table 4:** Logmar visual acuity comparing initial presentation and final follow-up GCA for normal and abnormal MRI orbital exams.

GCA (number of patients)	Normal orbital MRI (4)	Abnormal orbital MRI (9)	P value*
Initial presentation	1.038	1.125	0.84
Final follow up	1.202	1.135	0.89

*P value <0.05 denotes statistical significance.

## Discussion

Giant cell arteritis is an ophthalmological emergency with prompt diagnosis offering the best opportunity to avert visual loss (1). However, diagnosis remains challenging, and there continues to be a lack of clinical or laboratory definitive tests (4). Historically, imaging has focused on assessing the large vessels for signs of vasculitis or guiding biopsy. Recent advances in vessel wall imaging offer promise in detecting temporal artery inflammatory changes, mapping the extent of inflammation, guiding in a biopsy, and assessing the relationship of the vessel to the facial nerve (36–38). Intra-orbital imaging findings of GCA should be better studied and thoroughly investigated as potential markers for diagnosing GCA and distinguishing it from the clinically overlapping, differently managed NAION.

Our study assessed 13 patients with biopsy-proven GCA for intra-orbital findings with multiple exciting and potentially helpful imaging observations. First, detection of abnormal imaging findings is relatively common in GCA, with nine patients (69.2%) demonstrating at least one abnormal imaging finding on MR. Second, imaging findings are not confined to the symptomatic orbit but can also involve the non-symptomatic orbit, as four patients in this study demonstrated imaging abnormality in the noninvolved orbit. Third, the most common imaging finding was perineuritis with enhancement surrounding the intra-orbital optic nerve sheath complex without clinical optic nerve dysfunction. This was present in 8 patients, seven bilateral, including four asymptomatic orbits. Perineuritis can be explained by the involvement of the supplying vessels by the vasculitis, and the involvement of the asymptomatic orbit suggests an indirect sign of extension of the vasculitis to the orbit. Fourth, abnormal imaging findings are not isolated to the optic nerve sheath complex as two patients had optic disc edema and two additional patients had involvement of rectus muscles; in 1 case, this was the only abnormal imaging finding in the orbit. Our results align with a recently published multi-center study by Guggenberger et al. assessing intra-orbital imaging findings of GCA on black blood MRI (39). Guggenberger et al. noted 32% of patients with abnormal orbital imaging findings (18/56) (39). Similar to our study, the most common imaging finding was optic nerve sheath enhancement noted in 13 patients, with 12 being bilateral (39). However, unlike our study, Guggenberger et al. did not note any involvement of the extraocular muscles (39). Also, in Guggenberger et al. study optic neuropathy was almost nonexistent, with only one of 56 patients developing such involvement (39).

Another interesting aspect of our study was assessing the potential role of orbital MR imaging abnormalities as a diagnostic and predictive tool. Our study evaluated the relationship between clinical and laboratory data of abnormal and normal orbits on MRI for patients with GCA. We did not note a significant difference between the two groups. We also assessed both groups’ initial and final vision acuity and did not find significant differences. While the group sample is small (9 vs. four patients), preliminary exploratory data suggests the abnormal orbital findings might not correlate with laboratory/inflammatory data and might not serve as a predictor of long-term visual status. However, more extensive studies are needed to assess this further. In addition, it would be interesting to correlate these imaging findings with vessel wall imaging studies to map the association between vessel inflammation and orbital imaging findings.

Lastly, NAION and arteritic AION can overlap clinically, and we considered the possibility ofassessing the MR orbital findings of NAION and assessing these findings as a potential aid in differentiating between both entities. NAION orbital MR findings are under-reported in the literature. In our study, we noted multiple interesting imaging findings in NAION patients. First, all patients had abnormal orbital MR findings, suggesting that orbital imaging findings in this entity are often underreported. Second, in all patients, the abnormal MR imaging was unilateral, even in cases of subsequent visual loss, with MR demonstrating abnormal imaging of the initial symptomatic orbit only. Third, the optic nerve was involved in all cases, and the abnormality was hyperintensity of the optic nerve on fluid-sensitive sequences without associated enhancement.

The combination of these factors likely reflects the mechanism of non-vasculitic ischemia, and explains the lack of enhancement. The only additional imaging finding noted was optic disc edema, which was present in two of the eight patients.

This study suggests that orbital imaging findings may help distinguish between GCA and NAION, especially in cases of clinical uncertainty. It indicates that GCA is more likely to be bilateral, while NAION is typically unilateral. Also, GCA most commonly presents with the enhancement of the optic nerve sheath complex with sparring of the optic nerve. In contrast, NAION normally presents with abnormal optic nerve signal on a fluid-sensitive sequence without enhancement. Lastly, both typically involve the intra-orbital segment of the optic nerve with occasional associated optic disc edema.

Our study has a few limitations, including the retrospective nature of the study and the small sample size. However, in the case of GCA, this is offset by the inclusion of only biopsy-proven cases, and our study is one of the largest single-center studies assessing imaging findings of GCA. Also, including clinical, laboratory, and visual acuity data further strengthens the study and adds to the robustness of the data. Similarly, the NAION patient population is small, but robust inclusion criteria offset this. Also, NAION orbital imaging assessment needs to be more present in the radiology literature, and this article attempted to assess the imaging findings while correlating them with clinical, laboratory, and visual acuity data. Lastly, the article tried to assess imaging utility in aiding the clinician in distinguishing between the two often overlapping entities. Lastly, our study is exploratory for future collaborative studies in determining the relationship between vessel involvement and orbital findings in GCA and the relationship between imaging findings and visual outcomes.

## Conclusion

GCA is the most common vasculitis in patients above 50 years old, with the potential for visual loss. Our retrospective case series suggests that MRI orbital imaging can offer a potential clue for diagnosis, with most patients demonstrating imaging abnormalities. Optic nerve perineuritis is the most common imaging finding and is frequently bilateral. Our case series suggests that NAION, a potentially overlapping clinical syndrome poorly assessed in the neuroradiology literature, also often demonstrates imaging abnormalities that differ from GCA imaging findings. Optic nerve non-enhancing hyperintensity on fluid-sensitive sequence is the most common imaging finding on NAION. Lastly, our data suggests that abnormal imaging findings in GCA do not correlate with visual acuity outcomes. However, more extensive studies are needed to explore this further and correlate GCA imaging findings with vascular and clinical outcomes.

## Data Availability

The original contributions presented in the study are included in the article/supplementary material. Further inquiries can be directed to the corresponding author.

## References

[1] Diamantopoulos AP Haugeberg G Lindland A Myklebust G. The fast-track ultrasound clinic for early diagnosis of giant cell arteritis significantly reduces permanent visual impairment: towards a more effective strategy to improve clinical outcome in giant cell arteritis? Rheumatol (Oxford). (2016) 55:66–70. doi: 10.1093/rheumatology/kev289 26286743

[2] Li KJ Semenov D Turk M Pope J. A meta-analysis of the epidemiology of giant cell arteritis across time and space. Arthritis Res Ther. (2021) 23:82. doi: 10.1186/s13075-021-02450-w 33706808 PMC7948334

[3] Ponte C Martins-Martinho J Luqmani RA. Diagnosis of giant cell arteritis. Rheumatol (Oxford). (2020) 59:iii5–iii16. doi: 10.1093/rheumatology/kez553 32348512

[4] van der Geest KSM Sandovici M Brouwer E Brouwer E Mackie SL. Diagnostic accuracy of symptoms, physical signs, and laboratory tests for giant cell arteritis: A systematic review and meta-analysis. JAMA Intern Med. (2020) 180:1295–304. doi: 10.1001/jamainternmed.2020.3050 PMC7432275 32804186

[5] Prior JA Ranjbar H Belcher J Mackie SL Helliwell T Liddle J. Diagnostic delay for giant cell arteritis - a systematic review and meta-analysis. BMC Med. (2017) 15:120. doi: 10.1186/s12916-017-0871-z 28655311 PMC5488376

[6] Dejaco C Ramiro S Duftner C Bosch P Ponte C Mackie SL. EULAR recommendations for the use of imaging in large vessel vasculitis in clinical practice. Ann Rheum Dis. (2018) 77:636–43. doi: 10.1136/annrheumdis-2017-212649 29358285

[7] Breuer GS Nesher R Nesher G. Effect of biopsy length on the rate of positive temporal artery biopsies. Clin Exp Rheumatol. (2009) 27:S10–3. 19646339

[8] Ypsilantis E Courtney ED Chopra N Karthikesalingam A Eltayab M Katsoulas N. Importance of specimen length during temporal artery biopsy. Br J Surg. (2011) 98:1556–60. doi: 10.1002/bjs.7595 21706476

[9] Mahr A Saba M Kambouchner M Polivka M Baudrimont M Brochériou I. Temporal artery biopsy for diagnosing giant cell arteritis: the longer, the better? Ann Rheum Dis. (2006) 65:826–8. 10.1136/ard.2005.042770 PMC1798165 16699053

[10] Germanò G Muratore F Cimino L Lo Gullo A Possemato N Macchioni P. Is colour duplex sonography-guided temporal artery biopsy useful in the diagnosis of giant cell arteritis? A randomized study. Rheumatol (Oxford). (2015) 54:400–4. doi: 10.1093/rheumatology/keu241 24939678

[11] Luqmani R Lee E Singh S Gillett M Schmidt WA Bradburn M. The Role of Ultrasound Compared to Biopsy of Temporal Arteries in the Diagnosis and Treatment of Giant Cell Arteritis (TABUL): a diagnostic accuracy and cost-effectiveness study. Health Technol Assess. (2016) 20:1238. doi: 10.3310/hta20900 PMC5165283 27925577

[12] Guffey Johnson J Grossniklaus HE Margo CE Foulis P. Frequency of unintended vein and peripheral nerve biopsy with temporal artery biopsy. Arch Ophthalmol. (2009) 127:703. doi: 10.1001/archophthalmol.2009.77 19433728

[13] Murchison AP Bilyk JR. Brow ptosis after temporal artery biopsy: incidence and associations. Ophthalmology. (2012) 119:2637–42. doi: 10.1016/j.ophtha.2012.07.020 22986114

[14] Ing EB Wang DN Kirubarajan A Benard-Seguin E Ma J Farmer JP. Systematic review of the yield of temporal artery biopsy for suspected giant cell arteritis. Neuroophthalmology. (2018) 43:1825. 10.1080/01658107.2018.1474372 PMC6351023 30723520

[15] Maz M Chung SA Abril A Langford CA Gorelik M Guyatt G. 2021 American college of rheumatology/Vasculitis foundation guideline for the management of giant cell arteritis and takayasu arteritis. Arthritis Rheumatol. (2021) 73:1349–65. doi: 10.1002/art.41774 PMC12344528 34235884

[16] Schmidt WA Kraft HE Vorpahl K Völker L Gromnica-Ihle EJ. Color duplex ultrasonography in the diagnosis of temporal arteritis. N Engl J Med. (1997) 337:1336–42. doi: 10.1056/NEJM199711063371902 9358127

[17] Karassa FB Matsagas MI Schmidt WA Ioannidis JP. Meta-analysis: Test performance of ultrasonography for giant-cell arteritis. Ann Intern Med. (2005) 142:359–69. doi: 10.7326/0003-4819-142-5-200503010-00011 15738455

[18] De Miguel E Castillo C Rodríguez A De Agustín JJ. Working Group Ultrasound Giant Cell Arteritis Learning and reliability of colour Doppler ultrasound in giant cell arteritis. Clin Exp Rheumatol. (2009) 27:S53–8. 19646347

[19] Arida A Kyprianou M Kanakis M Sfikakis PP. The diagnostic value of ultrasonography derived edema of the temporal artery wall in giant cell arteritis: A second meta-analysis. BMC Musculoskelet Disord. (2010) 11:44. doi: 10.1186/1471-2474-11-44 20210989 PMC2837862

[20] Prieto-González S Arguis P García-Martínez A Espígol-Frigolé G Tavera-Bahillo I Butjosa M. Large vessel involvement in biopsy-proven giant cell arteritis: prospective study in 40 newly diagnosed patients using CT angiography. Ann Rheum Dis. (2012) 71:1170–6. doi: 10.1136/annrheumdis-2011-200865 22267328

[21] Lariviere D Benali K Coustet B Pasi N Hyafil F Klein I. Positron emission tomography and computed tomography angiography for the diagnosis of giant cell arteritis: A real-life prospective study. Medicine. (2016) 95:e4146. doi: 10.1097/MD.0000000000004146 27472684 PMC5265821

[22] De Boysson H Dumont A Liozon E Lambert M Boutemy J Maigné G. Giant-cell arteritis: Concordance study between aortic CT angiography and FDG-PET/CT in detection of large-vessel involvement. Eur J Nucl Med Mol Imaging. (2017) 44:2274–9. doi: 10.1007/s00259-017-3774-5 28736805

[23] Vaidyanathan S Chattopadhyay A Mackie SL Scarsbrook AF. Comparative effectiveness of 18FFDG PET-CT and contrast-enhanced CT in the diagnosis of suspected large-vessel vasculitis. Br J Radiol. (2018) 91:20180247. doi: 10.1259/bjr.20180247 29927635 PMC6223176

[24] Riemer HJAS Writing Group. Reviewer Group. Members of EANM Cardiovascular. Members of EANM Infection & Inflammation. Members of Committees. SNMMI Cardiovascular. Members of Council. PET Interest Group. Members of ASNC et al. FDG-PET/CT(A) imaging in large vessel vasculitis and polymyalgia rheumatica: Joint procedural recommendation of the EANM, SNMMI, and the PET Interest Group (PIG), and endorsed by the ASNC. Eur J Nucl Med Mol Imaging. (2018) 45:1250–69. doi: 10.1007/s00259-018-3973-8 PMC5954002 29637252

[25] Bley TA Uhl M Carew J Markl M Schmidt D Peter HH. Diagnostic value of high-resolution MR imaging in giant cell arteritis. AJNR Am J Neuroradiol. (2007) 28:1722–7. doi: 10.3174/ajnr.A0638 PMC8134183 17885247

[26] Geiger J Bley T Uhl M Frydrychowicz A Langer M Markl M. Diagnostic value of T2-weighted imaging for the detection of superficial cranial artery inflammation in giant cell arteritis. J Magn Reson Imaging. (2010) 31:470–4. doi: 10.1002/jmri.22047 20099359

[27] Klink T Geiger J Both M Ness T Heinzelmann S Reinhard M. Giant cell arteritis: Diagnostic accuracy of MR imaging of superficial cranial arteries in initial diagnosis-results from a multicenter trial. Radiology. (2014) 273:844–52. doi: 10.1148/radiol.14140056 25102371

[28] Klink T Geiger J Both M Carette S Clements-Baker M Cohen-Hallaleh V. High-resolution magnetic resonance imaging of scalp arteries for the diagnosis of giant cell arteritis: results of a prospective cohort study. Arthritis Rheumatol. (2017) 69:161–8. 10.1002/art.39824 27483045

[29] D'Souza NM Morgan ML Almarzouqi SJ Lee AG. Magnetic resonance imaging findings in giant cell arteritis. Eye (Lond). (2016) 30:758–62. doi: 10.1038/eye.2016.19 PMC4869132 26915748

[30] Morgenstern KE Ellis BD Schochet SS Linberg JV. Bilateral optic nerve sheath enhancement from giant cell arteritis. J Rheumatol. (2003) 30:625–7. 12610827

[31] Liu KC Chesnutt DA. Perineural optic nerve enhancement on magnetic resonance imaging in giant cell arteritis. J Neuroophthalmol. (2013) 33:279–81. doi: 10.1097/WNO.0b013e3182915b77 23845995

[32] Vodopivec I. Rizzo JF 3rd. Ophthalmic manifestations of giant cell arteritis. Rheumatol (Oxford). (2018) 57:ii63–72. 10.1093/rheumatology/kex428 29986083

[33] Viets R Parsons M Van Stavern G Hildebolt C Sharma A. Hyperintense optic nerve heads on diffusion-weighted imaging: a potential imaging sign of papilledema. AJNR Am J Neuroradiol. (2013) 34:1438–42. doi: 10.3174/ajnr.A3388 PMC8051484 23370477

[34] Holladay J. Proper method for calculating average visual acuity. J Refractive Surg. (1997) 13:388–91. doi: 10.3928/1081-597X-19970701-16 9268940

[35] Kaiser PK. Prospective evaluation of visual acuity assessment: a comparison of Snellen versus ETDRS charts in clinical practice (An AOS Thesis). Trans Am Ophthalmol Soc. (2009) 107:311–24. PMC2814576 20126505

[36] El Haddad J Charbonneau F Guillaume J Clavel G Chazal T Poillon G. Reproducibility and accuracy of vessel wall MRI in diagnosing giant cell arteritis: a study with readers of varying expertise. Eur Radiol. (2024). doi: 10.1007/s00330-023-10567-6 38206404

[37] Rheaume M Rebello R Pagnoux C Carette S Clements-Baker M Cohen-Hallaleh V. High-resolution magnetic resonance imaging of scalp arteries for diagnosis of giant cell arteritis: results of a prospective cohort study. Arthritis Rheumatol. (2017) 69:161–68. 10.1002/art.39824 27483045

[38] Poillon G Collin A Benhamou Y Clavel G Savatovsky J Pinson C. Increased diagnostic accuracy of giant cell arteritis using three-dimensional fat-saturated contrast-enhanced vessel-wall magnetic resonance imaging at 3T. Eur Radiol. (2020) 30:1866–75. doi: 10.1007/s00330-019-06536-7 31811430

[39] Guggenberger KV Vogt ML Song JW Weng AM Fröhlich M Schmalzing M. Intraorbital findings in giant cell arteritis on black blood MRI. Eur Radiol. (2023) 33:2529–35. doi: 10.1007/s00330-022-09256-7 PMC10017783 36394601

